# Supplementary material for: Microglial depletion rescues spatial memory impairment caused by LPS administration in adult mice
Source: PeerJ. 2024 Nov 15;12:e18552. doi: 10.7717/peerj.18552 (PMC11572354; doi:10.7717/peerj.18552)
Supplement: Supplemental Information 3 [file peerj-12-18552-s003.pdf]

ITEM TO CHECK	IMPORTANCE	CHECKLIST
**EXPERIMENTAL DESIGN**		
Definition of experimental and control groups	**E**	p14, line227-240
Number within each group	**E**	p27
Assay carried out by core lab or investigator's lab?	D	p10, line137-156
Acknowledgement of authors' contributions	D	p17,line293-295
**SAMPLE**		
Description	**E**	p10, line137-156
Volume/mass of sample processed	D	p10, line137-156
Microdissection or macrodissection	**E**	p10, line137-156
Processing procedure	**E**	p10, line137-156
If frozen - how and how quickly?	**E**	p10, line137-156
If fixed - with what, how quickly?	**E**	
Sample storage conditions and duration (especially for FFPE samples)	**E**	p10, line137-156
**NUCLEIC ACID EXTRACTION**		
Procedure and/or instrumentation	**E**	p10, line137-156
Name of kit and details of any modifications	**E**	p10, line137-156
Source of additional reagents used	D	p10, line137-156
Details of DNase or RNAse treatment	**E**	p10, line137-156
Contamination assessment (DNA or RNA)	**E**	p10, line137-156
Nucleic acid quantification	**E**	p10, line137-156
Instrument and method	**E**	p10, line137-156
Purity (A260/A280)	D	p10, line137-156
Yield	D	p10, line137-156
RNA integrity method/instrument	**E**	p10, line137-156
RIN/RQI or Cq of 3' and 5' transcripts	**E**	
Electrophoresis traces	D	
Inhibition testing (Cq dilutions, spike or other)	**E**	
**REVERSE TRANSCRIPTION**		
Complete reaction conditions	**E**	p10, line137-156
Amount of RNA and reaction volume	**E**	p10, line137-156
Priming oligonucleotide (if using GSP) and concentration	**E**	
Reverse transcriptase and concentration	**E**	p10, line137-156
Temperature and time	**E**	p10, line137-156
Manufacturer of reagents and catalogue numbers	D	p10, line137-156
Cqs with and without RT	D*	
Storage conditions of cDNA	D	p10, line137-156
**qPCR TARGET INFORMATION**		
If multiplex, efficiency and LOD of each assay.	**E**	
Sequence accession number	**E**	
Location of amplicon	D	p10, line137-156
Amplicon length	**E**	
In silico specificity screen (BLAST, etc)	**E**	
Pseudogenes, retropseudogenes or other homologs?	D	
Sequence alignment	D	
Secondary structure analysis of amplicon	D	
Location of each primer by exon or intron (if applicable)	**E**	
What splice variants are targeted?	**E**	
**qPCR OLIGONUCLEOTIDES**		
Primer sequences	**E**	p10, line137-156
RTPrimerDB Identification Number	D	
Probe sequences	D**	
Location and identity of any modifications	**E**	
Manufacturer of oligonucleotides	D	p10, line137-156
Purification method	D	
**qPCR PROTOCOL**		
Complete reaction conditions	**E**	p10, line137-156
Reaction volume and amount of cDNA/DNA	**E**	p10, line137-156
Primer, (probe), Mg++ and dNTP concentrations	**E**	
Polymerase identity and concentration	**E**	
Buffer/kit identity and manufacturer	**E**	p10, line137-156
Exact chemical constitution of the buffer	D	
Additives (SYBR Green I, DMSO, etc.)	**E**	p10, line137-156
Manufacturer of plates/tubes and catalog number	D	
Complete thermocycling parameters	**E**	p10, line137-156
Reaction setup (manual/robotic)	D	
Manufacturer of qPCR instrument	**E**	p10, line137-156
**qPCR VALIDATION**		
Evidence of optimisation (from gradients)	D	
Specificity (gel, sequence, melt, or digest)	**E**	p10, line137-156
For SYBR Green I, Cq of the NTC	**E**	p10, line137-156
Standard curves with slope and y-intercept	**E**	
PCR efficiency calculated from slope	**E**	
Confidence interval for PCR efficiency or standard error	D	
r2 of standard curve	**E**	
Linear dynamic range	**E**	
Cq variation at lower limit	**E**	
Confidence intervals throughout range	D	
Evidence for limit of detection	**E**	
If multiplex, efficiency and LOD of each assay.	**E**	
**DATA ANALYSIS**		
qPCR analysis program (source, version)	**E**	
Cq method determination	**E**	p10, line137-156
Outlier identification and disposition	**E**	
Results of NTCs	**E**	
Justification of number and choice of reference genes	**E**	p10, line137-156
Description of normalisation method	**E**	
Number and concordance of biological replicates	D	p10, line137-156
Number and stage (RT or qPCR) of technical replicates	**E**	
Repeatability (intra-assay variation)	E	p10, line137-156
Reproducibility (inter-assay variation, %CV)	D	p11, line170-174
Power analysis	D	p11, line170-174
Statistical methods for result significance	**E**	p11, line170-174
Software (source, version)	E	p11, line170-174
Cq or raw data submission using RDML	**D**	

Table 1. MIQE checklist for authors, reviewers and editors. All essential information (E) must be submitted with the manuscript. Desirable information (D) should be submitted if available. If using primers obtained from RTPrimerDB, information on qPCR target, oligonucleotides, protocols and validation is available from that source.

*: Assessing the absence of DNA using a no RT assay is essential when first extracting RNA. Once the sample has been validated as RDNA-free, inclusion of a no-RT control is desirable, but no longer essential.

**: Disclosure of the probe sequence is highly desirable and strongly encouraged. However, since not all commercial pre-designed assay vendors provide this information, it cannot be an essential requirement. Use of such assays is advised against.
